# Evaluation of the static and dynamic assistive torque of a passive upper limb occupational exoskeleton

**DOI:** 10.1017/wtc.2025.8

**Published:** 2025-04-15

**Authors:** Etienne Ricard, Chris Hayot, Isabelle Clerc-Urmès, Laurent Claudon, Kévin Desbrosses, Charles Pontonnier

**Affiliations:** 1 Département Sciences Appliquées au Travail et aux Organisations, Institut National de Recherche et Sécurité (INRS), Vandoeuvre-les-Nancy, France; 2 ComBO research team, Université de Rennes, Inria, CNRS, IRISA-UMR 6074, Rennes, France

**Keywords:** biomechanics, exoskeletons, human-robot interaction, performance characterization

## Abstract

Adjusting the assistive torque of upper limb occupational exoskeletons is essential to optimize their effectiveness and user acceptance in companies. This adjustment enables a balance to be struck between the expected benefits and potential undesirable effects associated with their use, particularly for the shoulder joint, which is sensitive to the balance of forces. Despite this, no study has yet evaluated these assistive torques in static and dynamic conditions representative of work situations. The aim of this article is therefore to evaluate these assistive torques under these two conditions, using an isokinetic dynamometer. Angular velocities ranging from 0 to 240°/s and four levels of assistance were investigated. The results showed that the maximum assistive torques in flexion (energy restitution phase) were lower than those in extension (tensioning phase) by 20 to 36% and were median in static conditions. It was also observed that the level of assistance and the exoskeleton opening angles had a strong impact on the assistive torques, unlike the angular velocity in dynamic conditions, which had a minimal effect. Quantifying these assistive torques is crucial for assessing their biomechanical impact and adjusting the exoskeleton’s assistance to the operator and the task performed.

## 1. Introduction

Passive upper limb exoskeletons (ULE) assisting the shoulder are mainly used for manual materials handling (MMH) and overhead work (OHW) tasks involving dynamic lifting or static holding of arms at height. In such situations, these technologies are designed to reduce muscular stress of the shoulders, which are the site of many musculoskeletal disorders (Barthelme et al., 2021). The operating principle of these exoskeletons is based on an external torque assisting the muscles involved in arm elevation. Many studies have evaluated the biomechanical effects of wearing ULE through experimental and modeling approaches (Moeller et al., 2022; Ma et al., 2023). These studies generally showed that the use of ULE significantly reduces the activity of the shoulder agonist muscles during arm elevation (Theurel and Desbrosses, 2019; Zhou and Zheng, 2021). However, there is considerable heterogeneity in the experimental results supposing a complex interaction between the exoskeleton, the user, and the task performed. Moreover, some studies reported the presence of undesirable effects: overactivations for shoulder antagonistic muscles in arm elevation, as well as for postural or rotator cuff muscles (Moeller et al., 2022; Kranenborg et al., 2023), excessive pressure at the human–exoskeleton interface (Linnenberg and Weidner, 2022), perceived discomfort, and limited ease of use (Kranenborg et al., 2023). Among the many hypotheses that could explain these observations (variations in effects and undesirable effects), the question of optimal assistance must be asked, both statically and dynamically. This is all the more true as the levels of assistance are rarely specified in previous studies, which does not allow for the impact of setting to be taken into account.

Conversely, some researchers have focused on the effect of adjusting the exoskeleton’s assistance in order to determine the best assistance setting. These studies have shown that increasing the level of assistance leads to a decrease in agonistic muscle activity and an increase in antagonistic muscle activity during shoulder elevation (Van Engelhoven et al., 2018; de Vries et al., 2019; Sänger et al., 2022). The adjustment of the assistance prevented an increase in shoulder reaction force (Sylla et al., 2014) or rotator cuff muscle activity (Seiferheld et al., 2022) and reduced the perception of effort (Van Engelhoven et al., 2018). The preferred settings varied depending on the weight handled and on the user perception (Sänger et al., 2022), underlining the importance of precise settings for each task and each user. Thus, the assistance level appears to be an important parameter that determines the exoskeleton effects, the mechanisms of which can only be studied if the quantification of assistance levels can be evaluated and modeled to match the requirements of the task. However, little is known about the mechanical behavior and recommended settings of each ULE in relation to specific task/morphology. Therefore, evaluating the assistive torques, and their variations during the movement (i.e., for different angles or angular velocities), seems to be a fundamental requirement to optimize the use of the exoskeleton. This knowledge would make it possible to adapt the assistive torque to the operator and the task carried out so that the exoskeleton is the most effective in terms of benefits, acceptance, and reducing undesirable effects.

Several studies have already proposed experimental approaches for evaluating the assistive torque of ULE and passive back exoskeletons. Methodologically, most of them have used load cells or force sensors (Koopman et al., 2019; Asgari et al., 2020; Hartmann et al., 2021; Van Harmelen and Schnieders, 2022; McCann et al., 2023). These studies provided information on the torques transmitted by exoskeletons to operators. Some of them reported higher torques during the tensioning phase of the exoskeleton assistance than during the restitution phase (i.e., hysteresis behavior). However, these studies have certain limitations. They are limited to low angular speeds, less than 20°/s, linked to customized test benches sensitive to vibration and with low acquisition frequencies. The position of the sensor depends on the experimenter and the design of the exoskeleton, making it difficult to reproduce these protocols. In addition, the reliability of the measurements has not been assessed. To overcome these limitations, other authors have characterized the exoskeleton torque–angle relationship using an isokinetic dynamometer. This device was used to characterize the assistive torques of two back exoskeletons in static and dynamic conditions, also highlighting hysteresis behavior (Madinei et al., 2022). However, for ULE, only static conditions were evaluated and modeled using polynomial equations (Watterworth et al., 2023), though most MMH situations involve dynamic movements.

Thus, this study aims to evaluate and model the assistive torque of an ULE using an isokinetic dynamometer in static and dynamic conditions. In particular, the study seeks to answer two questions (Q):

Q1: What assistive torque does the exoskeleton provide as a function of its settings (assistance level) and conditions of use (exoskeleton arm elevation angle, angular velocity and rotation direction of the movement)?

Q2: How to model these assistive torque profiles using equations, to integrate them in the design phase of a workstation? Knowing and modeling exoskeleton assistance as a function of setting and joint configuration has two major benefits. The first is to be able to propose optimal conditions of use for a given set of tasks. The second is to use the modeling in motion analysis tools to understand and quantify the biomechanical impact of the system on operator task performance.

## 2. Material and methods

### 2.1. Upper limb exoskeleton

**Figure 1.**
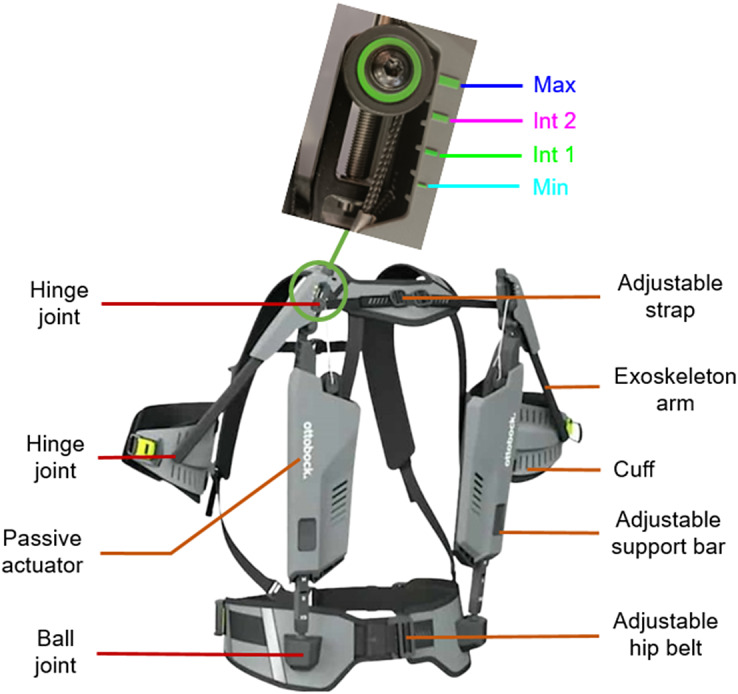
Ottobock shoulder exoskeleton with its different elements and assistance levels to the study: minimum in light blue (Min), two intermediate levels in green and magenta (Int 1 and Int 2), and a maximum in dark blue (Max) (Exoskeleton Report, 2022).

The Ottobock shoulder (Ottobock SE & Co. KGaA, Duderstadt, Germany) was assessed in this study (Figure 1). It is a passive ULE designed to assist the user during OHW tasks. It weighs 2 kg and assists elevation of each operator arm with a system composed of a bungee cord (passive actuator) and rigid segments (exoskeleton arm and adjustable support bar). The passive actuator is put under tension with the weight of the user’s arm or when lowering the exoskeleton arm. This system generates a torque depending on the elevation angle of the operator arm (Finke et al., 2022). The angular range of motion of the exoskeleton arm is 155°. Assistance level is adjustable by an endless screw and four levels of assistance are indicated as shown in Figure 1: a minimum (Min), two intermediate (Int1 and Int2), and a maximum level (Max). These four levels of assistance were tested in this study. To follow the kinematics of the shoulder, an adjustable support bar is connected to a hip belt with a ball joint. The adjustable support bar was mounted parallel to the passive actuator and is connected to the exoskeleton arm by a hinge joint. Finally, a cuff links the operator’s arm to the exoskeleton arm. The structure can be adapted for operators between 1.60 m and 1.90 m tall.

### 2.2. Isokinetic dynamometer

**Figure 2.**
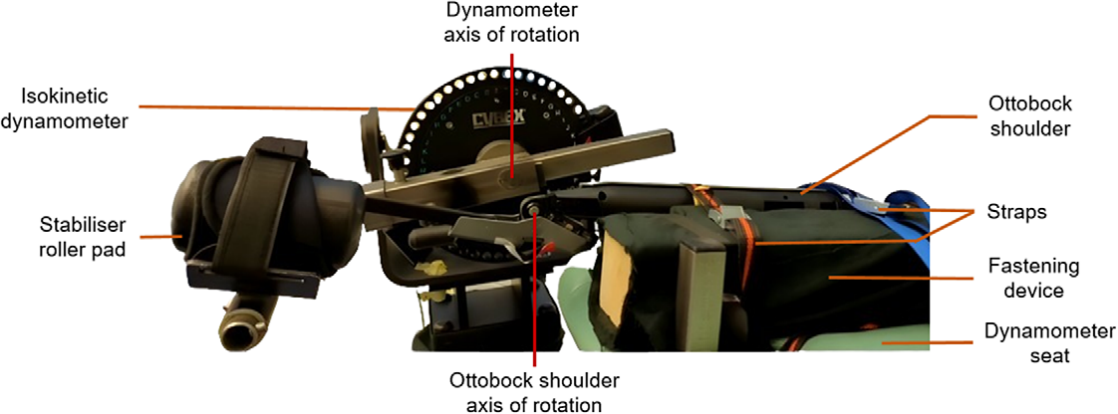
Detailed view of the test bench with the Ottobock shoulder exoskeleton positioned in its maximum opening angle (155°) on the HUMAC NORM isokinetic dynamometer.

The exoskeleton torque–angle relationship was quantified using the isokinetic dynamometer HUMAC NORM (Computer Sports Medicine, Inc., Stoughton, MA, USA) in isometric mode (for static conditions) and in continuous passive motion mode (for dynamic conditions) at different angular velocities. In this latter, the angular velocities were regulated by the integrated servo-motor of the isokinetic dynamometer. HUMAC NORM was validated to evaluate torque for both conditions in many studies (de Araujo Ribeiro Alvares et al., 2015; Habets et al., 2018; Estrázulas et al., 2020). The device settings were defined using the HUMAC 2015 software. The continuous passive motion mode was set with a cushion value of 0 corresponding to the slowest deceleration (softest stop) to avoid hard stop at the amplitude limits. The isokinetic dynamometer was attached to the exoskeleton’s cuff equipped with a stabilizer roller pad, as if it was a human arm. In addition, the rotation axes of the isokinetic dynamometer and the exoskeleton were visually aligned by means of a laser. The exoskeleton was securely fastened to the dynamometer seats using straps to prevent any unwanted movement (Figure 2). The torque, angle, and angular velocity data were recorded via the analog output of the HUMAC NORM and sampled at 1200 Hz.

### 2.3. Experimental protocol

Only one experimenter performed the measurements. In static condition (STA), the exoskeleton assistive torque was recorded over a period of 5 sec for a first measurement at 0° and for every 10° from 5 to 155° (i.e., 17 measuring angles). Five repetitions were performed for each angle and each of the four exoskeleton assistance levels. In dynamic conditions, the torque was continuously recorded during the movement of the exoskeleton arm over its full range of motion in both directions: from 0 to 155°, representing a shoulder flexion movement (FLE) if the exoskeleton was worn by a person, and from 155 to 0°, representing a shoulder extension movement (EXT). The positioning of the exoskeleton on the isokinetic dynamometer, as well as the corresponding positioning on an operator, are described in Supplementary Material A. Angular velocities ranging from 20 to 240°/s with an incremental step of 20°/s (i.e., 12 angular velocities) were completed. Five repetitions of flexion/extension movements were successively performed for each angular velocity and each of the four exoskeleton assistance levels. A pause of 2 sec was set between flexion and extension motion. Additionally, to isolate the exoskeleton torque from the net weight of the dynamometer arm, repetitions were carried out without the exoskeleton for each condition. During this protocol, angles, angular velocities, and levels of assistance were randomized.

### 2.4. Data processing

In order to determine the assistive torques provided by the exoskeleton, the raw data were low-pass filtered (fourth order bidirectional Butterworth filter, cut-off frequency = 3 Hz). The cut-off frequency was determined using the residual analysis method (Winter, 2009). For STA torques, each trial was averaged over 5 sec. For FLE and EXT torques, each trial was averaged every degree (from 0 to 1° and 154 to 155°). Therefore, the torque value corresponding to 0° is the average of the torque recorded within the interval [0°; 1°] and so on. For this reason, torque profiles were calculated for the static and dynamic conditions from 0 to 154°. The repeatability of the trials without exoskeleton under static and dynamic conditions, respectively, was checked using Bland–Altman diagrams (Martin Bland and Altman, 1986) to average these trials of each condition. These averages were subtracted from the total torque measured in the corresponding condition with exoskeleton. To compare the maximum torques and their positions in STA with FLE and EXT, cubic spline interpolation was performed on the 17 angles of isometric measurements. The data processing was carried out using Matlab (version R2023b).

### 2.5. Statistical analysis

The results were presented as the mean 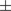 standard deviation for the five STA trials, with FLE and EXT for the four levels of assistance. FLE and EXT were presented for the 12 angular velocities. To compare each level of assistance, angular velocity and STA, FLE, and EXT conditions, a *T* test or a Wilcoxon rank sum test was used on the maximum torque and their positions, as appropriate. To determine assistive torque equations as a function of the considered opening angle, the level of assistance and angular velocity, according to the profiles considered for each of the STA, FLE, and EXT conditions, an ANOVA model was first performed. Main effects and their interactions were tested. Significant effects were then analyzed using Bonferroni *post hoc* comparison tests. The effect size was evaluated by means of Cohen’s *d* values for significant *post hoc* comparisons. The effect size was considered small ( 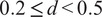 ), medium ( 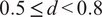 ), or large ( 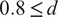 ) according to Cohen (1992). Only results with very small effect size ( 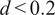 ) were not considered.

Then, piecewise polynomial regression models were used to take into account the STA, FLE, and EXT profiles and conditions (Ostertagová, 2012; Watterworth et al., 2023). Each regression model was evaluated at polynomial orders from 1 to 3. Following preliminary work to improve the adjusted *R*^2^ ( 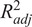 ) by 1% per added parameter, a maximum of six parameters were fixed. These models were selected with the highest 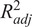 and the lowest Akaike information criterion (AIC) (Akaike, 1974; Sugiura, 1978; Hurvich et al., 1998; Wagenmakers and Farrell, 2004). Additionally, the minimal negative values of assistive torque obtained were utilized as indicators for model selection (Looft and Law, 2015).

Alpha risk was fixed to 5% for all analyses. The statistical analysis was carried out using STATA software (Stata 18, StataCorp., College Station, TX, USA).

## 3. Results

### 3.1. Exoskeleton assistive torque profiles

**Figure 3.**
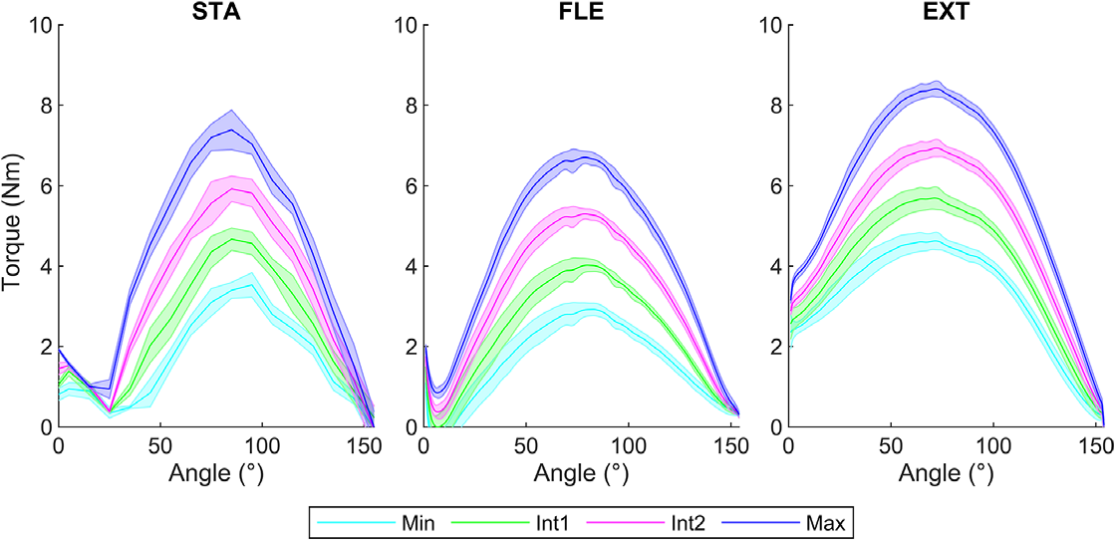
Torque–angle relationship of the Ottobock shoulder under different conditions: STA (left), FLE (center), and EXT (right) for the four assistance levels (Min, Int1, Int2, Max), represented by the mean with the standard deviation in the band. The dynamic conditions FLE and EXT are represented for the 12 angular velocities from 20 to 240°/s.

A descriptive presentation of the exoskeleton torque profile according to the exoskeleton opening angles for the four levels of assistance is shown in Figure 3. Bell-shaped curves can be observed. In STA, two profiles are identified on angular ranges [0°; 25°] and ]25°; 154°]. In FLE (all angular velocities combined), two profiles are identified on angular ranges [0°; 7°] and ]7°; 154°] and in EXT (all angular velocities combined), one profile is identified on the full angular range [0°; 154°].

### 3.2. Effect of assistance levels, velocity, and rotation direction

According to the previously defined statistical criteria, no effect of angular velocity in FLE on maximum torques and their positions was considered ( 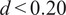 ). However, in EXT, the maximum torque decreased with faster angular velocities. Small effects size were obtained, from 80 to 240°/s compared to 20°/s ( 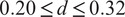 ), from 120 to 140°/s compared to 40°/s ( 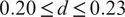 ), and for 120°/s compared to 60°/s (*d* = 0.20). For angular positions, the positions of the maximum torque at 240°/s were greater ( 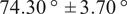 ) than those obtained for 100 ( 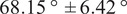 ) and 120°/s ( 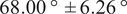 ) with large effects size ( 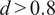 ).

**Figure 4.**
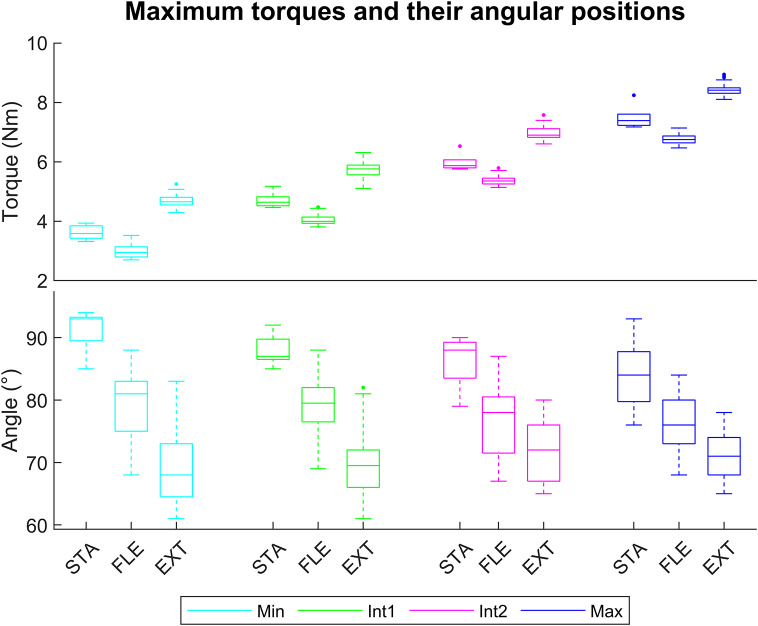
Boxplot representation to compare the exoskeleton’s maximum torques (top) and their angular positions (bottom) for the four assistance levels (Min, Int1, Int2, and Max) in STA, FLE, and EXT including 5 trials and 12 angular velocities.

Raising the assistance levels resulted in an increase in the maximum torques for STA, FLE, and EXT conditions, with large effect size ( 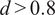 ) (Figure 4). However, it did not affect the angular positions for STA and EXT. But in FLE, there was a decrease in angular positions of maximum torque with a small effect size between Min and Max (*d* = 0.48), Int1 and Int2 (*d* = 0.49), and a moderate effect between Min and Int2 (*d* = 0.51) (Figure 4).

Concerning the maximum torque values, greater values were observed in EXT than in STA ( 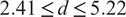 ), and in STA than in FLE ( 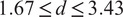 ) for each level of assistance and angular velocity, as depicted in Figure 4.

Concerning the position of these maximum torques, greater angles were obtained in STA than in FLE ( 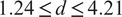 ), except for the Max level for angular velocities from 40 to 100°/s, and in FLE than in EXT ( 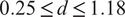 ) for a part of level of assistance and angular velocities, as shown in Figure 4.

### 3.3. Modeling of exoskeleton assistive torque

For the complete profiles in EXT, angular velocities had no significant effect. In FLE, there was a significant effect with a very small effect size where *d* < 0.2, so the angular velocity was not taken into account for modeling. For STA, FLE, and EXT, only the level of assistance had a large effect size when there was a difference of at least two levels of assistance ( 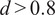 ). To model the assistive torques, two independent variables were used, where “*x*” corresponds to the exoskeleton opening angles from 0 to 154° and “*y*” to the levels of assistance between 1 and 4, where 1 is the Min level, 2 and 3 two intermediate levels, and 4 the Max level. To model STA, two equations were used: STA 1 on an angular range [0°; 25°] and STA 2 on ]25°; 154°]. In FLE, two equations were used: FLE 1 on [0°; 7°] and FLE 2 on ]7°; 154°]. In EXT, one equation was used, EXT 1 on [0°; 154°]. These equations are shown in Table 1 with good fitting indicators, root mean squared error (RMSE) between [0.16 Nm; 0.43 Nm] and 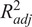 [0.882; 0.982]. The representation of these modeling results are provided in Supplementary Material B.

**Table 1.** Ottobock shoulder assistive torques equations fitted by polynomial regression models for STA, FLE, and EXT conditions. The equations were *z* = *f*(*x*,*y*), where *x* corresponds to the considered angle and *y* the assistance levels between 1 and 4, where 1 is the Min level, 2 and 3 Int1 and Int2 levels, and 4 the Max level, with their fitting indicators, RMSE and 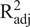

Conditions	Ottobock Shoulder assistive torque equations	RMSE (Nm)	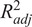	AIC
STA 1 [0°; 25°]	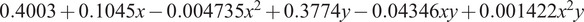	0.16	0.88	−65.11
STA 2 ]25°; 154°]	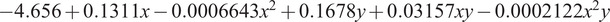	0.41	0.96	284.38
FLE 1 [0°; 7°]	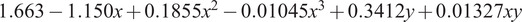	0.18	0.93	−949.24
FLE 2 ]7°; 154°]	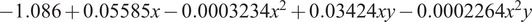	0.30	0.97	15834.35
EXT 1 [0°; 154°]	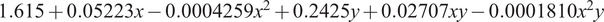	0.27	0.98	9461.19

## 4. Discussion

### 4.1. Assessment of exoskeleton assistive torque

The first objective of this study was to evaluate the assistive torque provided by an ULE under static and dynamic conditions.

In static conditions, the torque-opening angle relationships formed bell-shaped profiles. Maximum torque values ranged from 3.3 to 8.2 Nm, depending on the level of assistance, for opening angles between 79 and 94°. These results were similar to the assistive torques provided by the first version of the Ottobock shoulder (i.e., Paexo shoulder) with two modes, single spring and double spring. The maximum assistive torques varied from 3 to 6 Nm for the single spring mode, and from 6 to 11 Nm for the double spring mode. These torques were obtained for opening angles between 90 and 100°, using an isokinetic dynamometer under static conditions (Watterworth et al., 2023). The results of our study are also similar to other ULE for which the maximum torques varied between 4 and 13 Nm (Watterworth et al., 2023).

In dynamic conditions, the torque-opening angle relationships also formed bell-shaped profiles. Their maximum torques ranged between 2.7 and 8.9 Nm (according to the level of assistance) at corresponding opening angles between 60 and 85°. To our knowledge, there are no other studies in the literature reporting maximum dynamic assistive torques for ULE.

The angular velocities investigated in this study had minimal effect on the assistive torques. Similarly, previous research by Madinei et al. (2022) observed no effect on provided torque for two back exoskeletons across angular velocities ranging from 20 to 100°/s. This suggests that exoskeleton assistive torques are not significantly influenced by angular velocity, unlike the torque-producing capacity of the shoulder. In fact, the concentric torque production capacity at the shoulder decreased with increasing angular velocity (Otis et al., 1990; Whitcomb et al., 1995). The reported reductions were attributed to the muscle force–velocity relationship. Thus, the exoskeleton’s assistive torque could be more beneficial at higher velocity.

The rotation direction of the movement was also investigated in this study. The assistive torques were larger during the extension movement than the flexion movement and median in static. These results, comparing static and dynamic conditions, were also observed for two passive back exoskeletons (Madinei et al., 2022). These differences between static and dynamic conditions are mainly due to the friction forces of the device. These forces were subtracted from the static torques during the restitution phase (FLE) and added to them during the tensioning phase (EXT). In addition, the mechanical characteristics of the elastic band or spring lead to a loss of energy during the restitution phase. This difference in dynamic torque is known as hysteresis. Many studies refer to it for ULE (Asgari et al., 2020; McCann et al., 2023) or back exoskeletons (Koopman et al., 2019; Madinei et al., 2022). Maximum percentages of energy loss have been referenced from 28 to 51% for two back exoskeletons (Van Harmelen and Schnieders, 2022) and 28% for an ULE (Rossini et al., 2021). The results of this study indicated maximum energy losses of 36, 29, 23, and 20% for Min, Int1, Int2, and Max, respectively. The difference between FLE and EXT is constant at around 1.6 Nm. Compared with the torque production capacity of the shoulder, these results point in the same direction. For the same shoulder elevation angle, the torques generated are higher for an extension movement than for a flexion movement and median in static condition (Koski and McGill, 1994; Harbo et al., 2012).

Inverse bell-shaped profiles with low assistive torques were obtained on STA [0°; 25°] and FLE [0°; 7°] conditions. These profiles were also observed for the first version of the Ottobock shoulder (i.e., Paexo shoulder) and for other ULE (Watterworth et al., 2023). They result from the maximum tension of the force transmission cable when the opening angle is low. Indeed, in this configuration, the tension of the elastic does not allow the system to passively maintain itself at 0°. Therefore, there is a torque greater than 0 Nm at this angle. Then, the temporary reduction in torque for the following angles is probably due to the configuration of the pulleys designed to assist at the desired angle (Rossini et al., 2021; Kim et al., 2022). According to Kim et al., 2022, they enable permanent contact to be maintained between the exoskeleton cuff and the user’s arm.

### 4.2. Modeling of exoskeleton assistive torque

The second objective of this study was to model assistive profiles obtained during the evaluation of the assistive torques. The assistive profiles were modeled as a function of two selected input parameters, the opening angle of the exoskeleton “*x*” and the setting of its level of assistance “*y.*” In static conditions, piecewise polynomial modeling was performed to fit the two assistance profiles. These equations are STA 1 for angles ranging from [0°; 25°] and STA 2 for angles ranging from ]25°; 154°]. These models have high 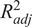 values of 0.88 and 0.96, respectively, attesting to the accurate modeling of the assistance profiles. To our knowledge, Watterworth et al. (2023) is the only study to have modeled the assistive torque of ULE as a function of the shoulder angle elevation in static condition. An equation per level of assistance was performed with similar 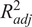 . They hypothesized that the shoulder elevation angle was close enough to the exoskeleton opening angle to be included in the equation. Conversely, in this study, if the opening angle of the exoskeleton is not known, it is possible to use the shoulder elevation angle as an input by making the same assumption. These equations will enable us to obtain the output assistive torques for a given level of assistance and angle in static conditions.

In dynamic conditions, a piecewise polynomial modeling was performed for FLE to fit the two assistance profiles, FLE1 and FLE2 on the angular ranges [0°; 7°] and ]7°; 154°], respectively. One equation was performed for EXT, EXT1 over the entire profile. These equations integrate the mechanical properties of the ULE under dynamic conditions for angular velocities of 20 to 240°/s corresponding to the majority of MMH tasks and part of the OHW tasks. Despite taking into account all the angular velocities, the fitting is very good with 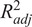 between [0.93; 0.98]. This study is the first to model exoskeleton assistive profiles under dynamic conditions. These five equations obtained in static and dynamic conditions can be used to obtain the assistive torques generated by the ULE during a work situation.

In the future, this work could help to adjust exoskeleton assistance according to the operator and the task. Based on these equations, it would be possible to develop a computer tool to adjust the exoskeleton’s assistance according to the shoulder torque for different work situations and anthropometry of operators (de Leva, 1996). Another approach for researchers would consist of estimating the effects of the ULE via digital human modeling (van der Have et al., 2022; Delgado-Llamas et al., 2023). Various assistive torques could be applied to the human biomechanical model at the shoulder. Thus, using a calculation procedure (inverse dynamics for example), it would be possible to calculate the forces at the shoulder joint and optimize the level of assistance to minimize joint stresses. In addition, a constrained optimization procedure could be used to calculate muscle forces and joint contact forces. These estimated musculoskeletal loads should enable an optimal balance to be found between the beneficial and undesirable effects of wearing the exoskeleton (Seiferheld et al., 2022). Current modeling approaches do not take into account the mechanical behavior of exoskeletons, such as hysteresis (Ma et al., 2023; Scherb et al., 2023). The protocol proposed in this study could be a way of improving the accuracy of digital human modeling and thus enabling the simulation of the exoskeleton effect during the design phase of a workstation.

Some limitations of this study should also be mentioned. The ULE was characterized under optimal conditions of use, with perfect alignment with the rotation axes of the isokinetic dynamometer. Real operating usage conditions do not guarantee perfect assistance efficiency, and the full assistive torque is not transmitted to the operator. The conditions of use may entail misalignments due to the system’s freedom of movement, slippages at the interface, contact forces, and so forth. In addition, the transfer of force from the assist torque to the pelvis was not measured and therefore could not be modeled. Furthermore, the protocol of this study included only one model of this ULE. However, it could be possible that differences in torque generation could exist between the same ULE models when they are manufactured. The reproducibility of this protocol for other exoskeletons could be limited by their design, particularly for exoskeletons with many degrees of freedom.

### 4.3. Practical application

ULE were designed to compensate for shoulder elevation torques through a maximum assistive torque at approximately 90°. Indeed, raising an outstretched arm with a 2.25 kg tool also generates a bell-shaped torque with a maximum value between 16 and 24 Nm (depending on the operator’s morphology) at 90° (Van Engelhoven et al., 2019). Furthermore, the shoulder’s maximum torque production capacity in flexion is at 0° (Otis et al., 1990; Koski and McGill, 1994). Thus, ULE supports the shoulder where it is most needed. However, if the elbow is flexed, the shoulder torque is reduced and the profile changes. For an elbow flexed at 90°, the maximum shoulder torque was obtained at 60° (Kim et al., 2022). Taking the example of a crate lifting task, the torque–angle relationship of exoskeletons was most effective for peak torques occurring between 75 and 105° (Seiferheld et al., 2022).

However, adjusting the level of assistance of ULEs remains challenging because the shoulder torques vary depending on the task. For overhead drilling tasks with a drill weighting about 2 kg, average shoulder torques of 10 Nm with a 90° elbow, and 30 Nm with an extended elbow were calculated (Anton et al., 2001). For another drilling task with a 2-kg tool, shoulder torques between 6 and 11 Nm were estimated (Panariello et al., 2022). For two-handed lifting tasks with weights of 5, 10, and 15 kg, the 95th percentiles of shoulder flexion torques were 18.7, 31.7 and 44.3 Nm respectively, and 19.7 Nm for one-handed OHW lifting tasks with a weight of 3 kg (Huysamen et al., 2020). The setting of the assistance could therefore be specific to the type of task (movement, weight handled, etc.). This specific setting may not be suitable for a sequence of tasks carried out in an occupational environment. A balance must be found between insufficient and excessive assistance (Sylla et al., 2014; Van Engelhoven et al., 2018; Sänger et al., 2022). A recent study recommended two different settings for static and dynamic pointing tasks. A maximum assistance (i.e., 6 Nm) was recommended for static tasks and a moderate level (60 or 80% of the maximum) for dynamic tasks so as not to increase the activation of antagonist muscles when lowering the arm (Ramella et al., 2024). The operation of ULE can be counterproductive for shoulder extension movements due to the opposing torque and hysteresis of these devices. Consequently, these devices should be used within their angular ranges of assistance for tasks involving holding the arms at shoulder height or overhead lifting. It is also essential to consider the length of time for which the ULE provides appropriate assistance. Furthermore, a potential solution to address the issues related to adjusting the level of assistance could involve real-time modulation through active or semiactive systems (Grazi et al., 2020; Crea et al., 2021).

## 5. Conclusion

The characterization of the ULE torques under static and dynamic conditions can be used to adapt the settings of the exoskeleton according to the individual characteristics of the user and the mechanical characteristics of the tasks. Going further toward individualized settings, the mechanical properties of the exoskeleton’s assistive torques, such as hysteresis, were modeled using equations with the exoskeleton’s assistance level and opening angle as inputs. In this way, the optimal setting can be assessed by simulation, striking a balance between the benefits and undesirable effects of wearing ULE.

## Supporting information

Ricard et al. supplementary material 1 Ricard et al. supplementary material

Ricard et al. supplementary material 2 Ricard et al. supplementary material

## Data Availability

The datasets generated during and/or analyzed during this study are available from the corresponding author on reasonable request.

## Supplementary material

The supplementary material for this article can be found at http://doi.org/10.1017/wtc.2025.8.
