# Supplementary material for: Evaluation of the static and dynamic assistive torque of a passive upper limb occupational exoskeleton
Source: Wearable Technol. 2025 Apr 15;6:e19. doi: 10.1017/wtc.2025.8 (PMC12034577; doi:10.1017/wtc.2025.8)
Supplement: Ricard et al. supplementary material 1 — Ricard et al. supplementary material [file S2631717625000088sup001.zip › CUP_Logo.pdf]

CAMBRIDGE
UNIVERSITY PRESS
