# Supplementary figures and images for: Evaluation of the static and dynamic assistive torque of a passive upper limb occupational exoskeleton
Source: Wearable Technol. 2025 Apr 15;6:e19. doi: 10.1017/wtc.2025.8 (PMC12034577; doi:10.1017/wtc.2025.8)

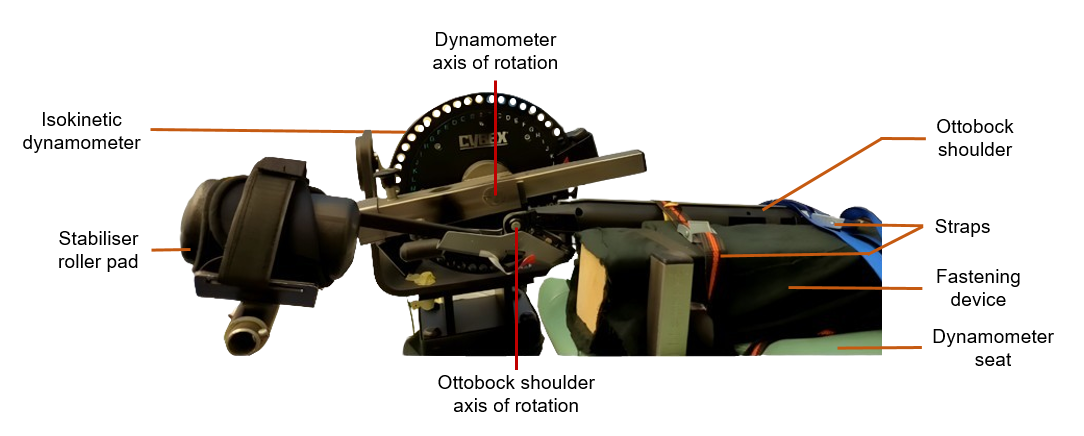

Supplement: Ricard et al. supplementary material 1 — Ricard et al. supplementary material [file S2631717625000088sup001.zip › Bench_Figure 2.PNG]

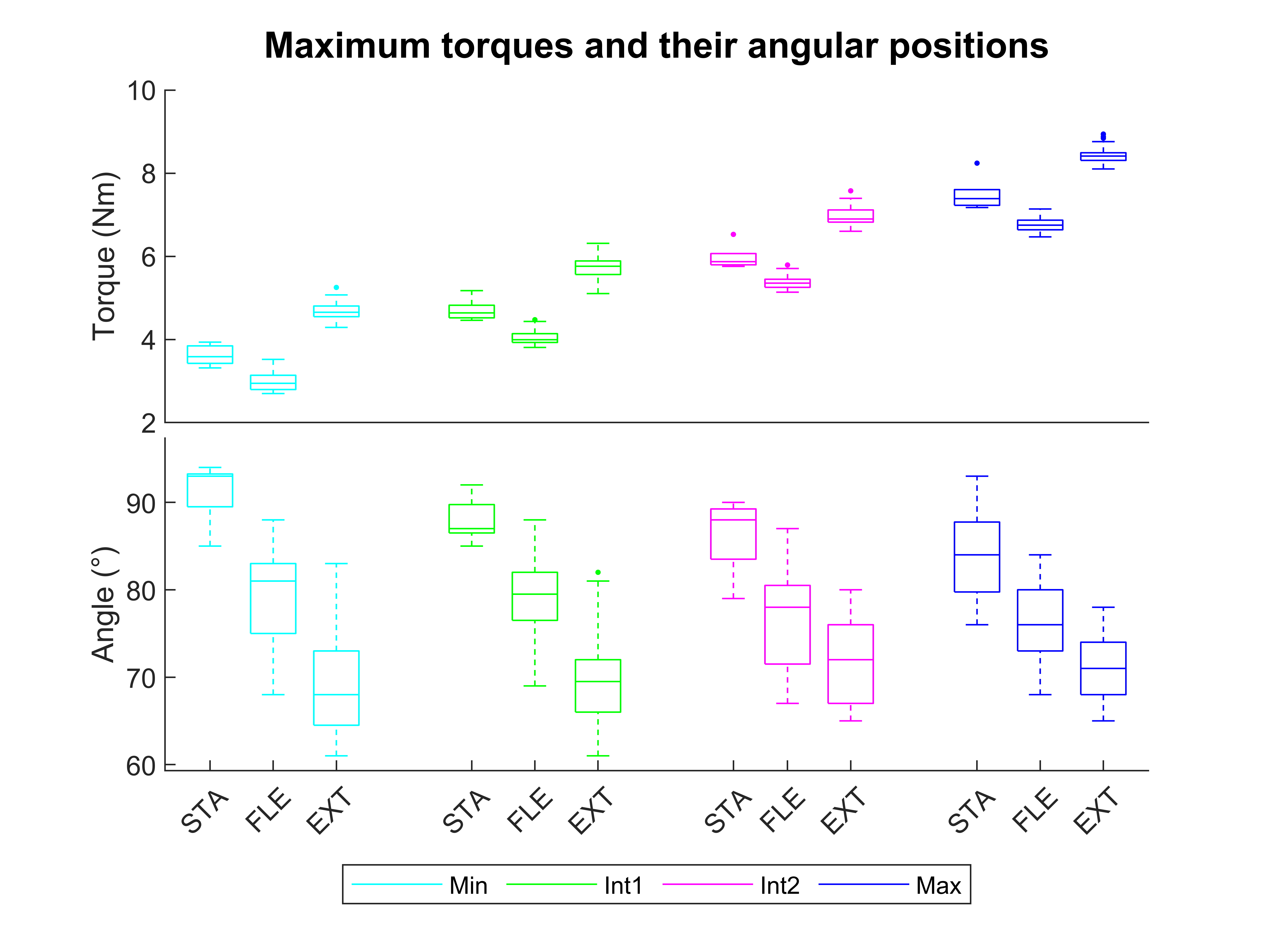

Supplement: Ricard et al. supplementary material 1 — Ricard et al. supplementary material [file S2631717625000088sup001.zip › Boxplot_Figure 4.png]

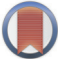

CrossMark

Supplement: Ricard et al. supplementary material 1 — Ricard et al. supplementary material [file S2631717625000088sup001.zip › CrossRef-logo.pdf]

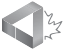

CMS  

---

SMC

Supplement: Ricard et al. supplementary material 1 — Ricard et al. supplementary material [file S2631717625000088sup001.zip › DataMath_Logo.pdf]

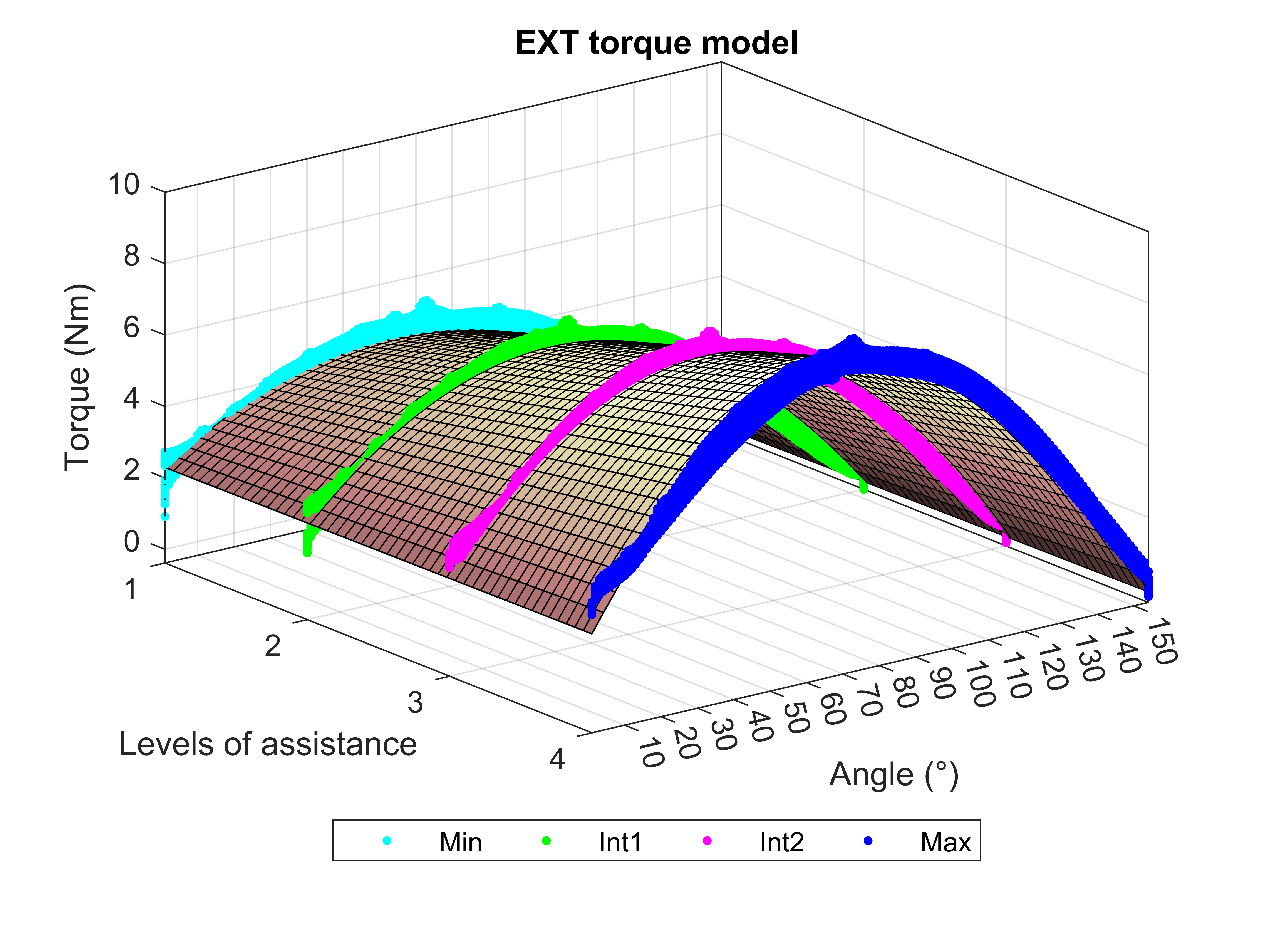

Supplement: Ricard et al. supplementary material 1 — Ricard et al. supplementary material [file S2631717625000088sup001.zip › EXT.png]

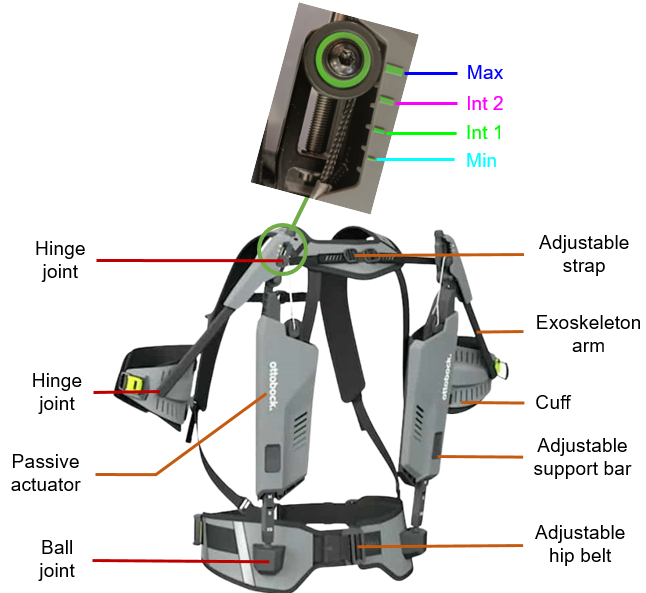

Supplement: Ricard et al. supplementary material 1 — Ricard et al. supplementary material [file S2631717625000088sup001.zip › Exo_Figure 1.png]

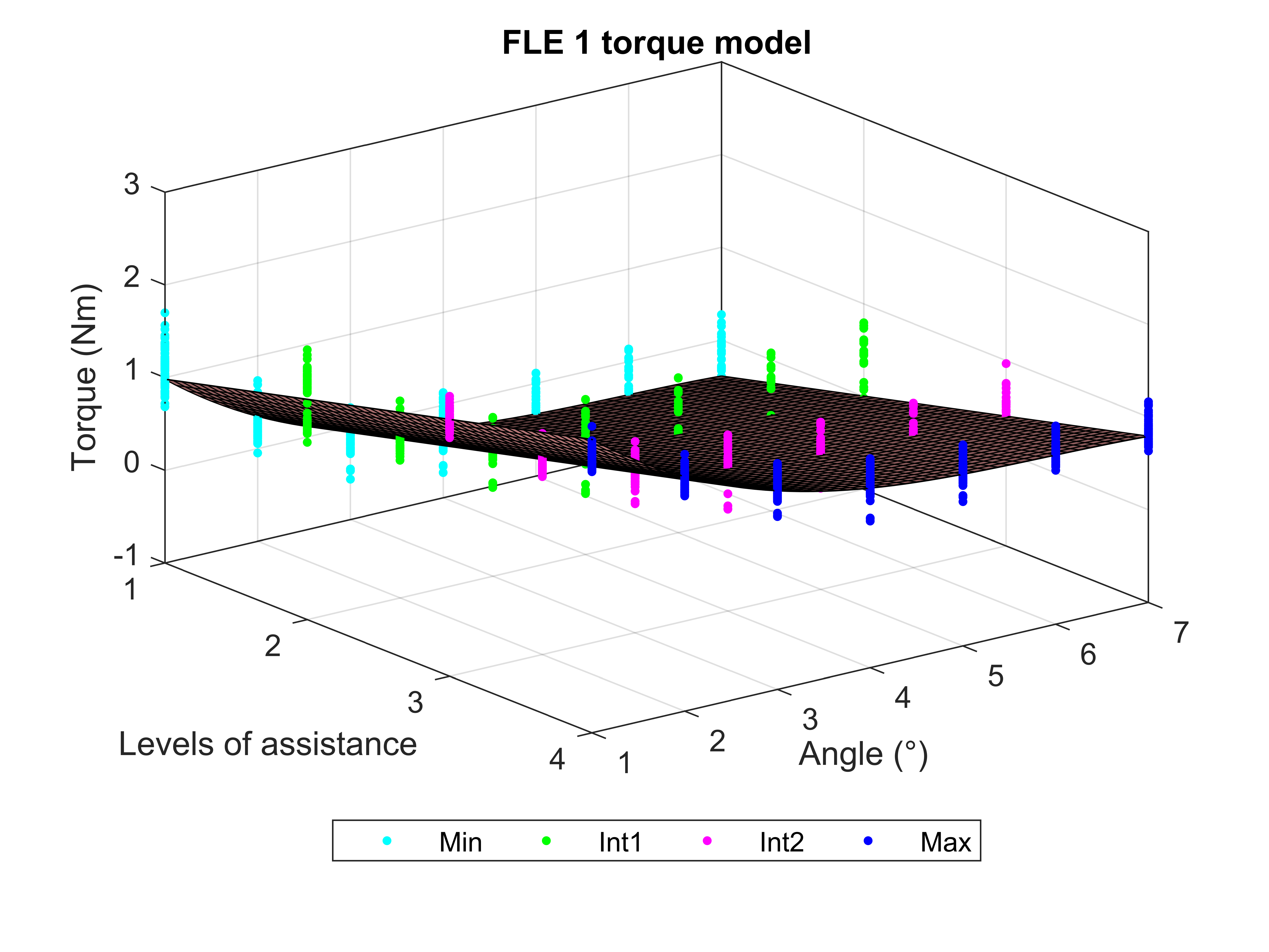

Supplement: Ricard et al. supplementary material 1 — Ricard et al. supplementary material [file S2631717625000088sup001.zip › FLE1.png]

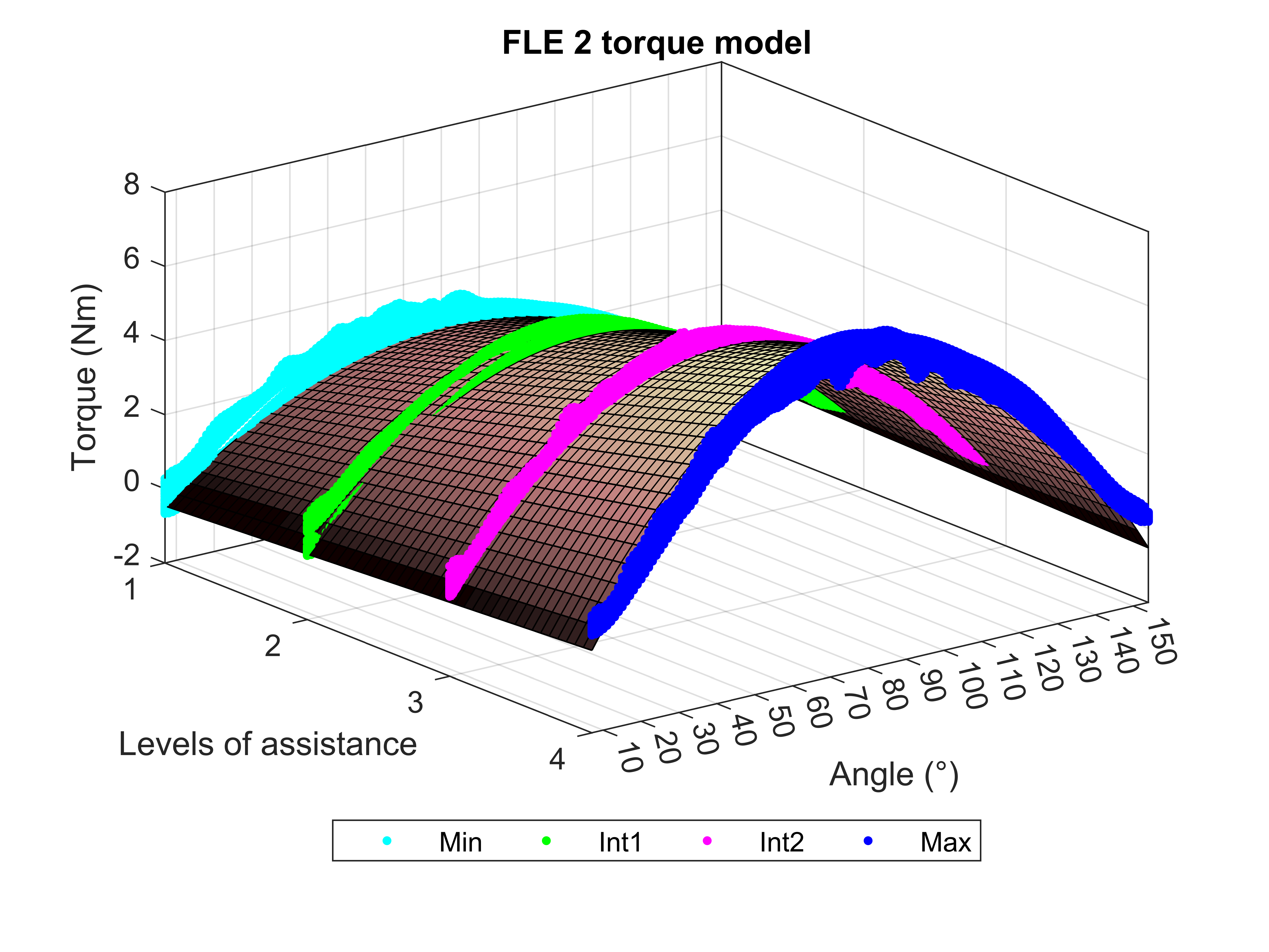

Supplement: Ricard et al. supplementary material 1 — Ricard et al. supplementary material [file S2631717625000088sup001.zip › FLE2.png]

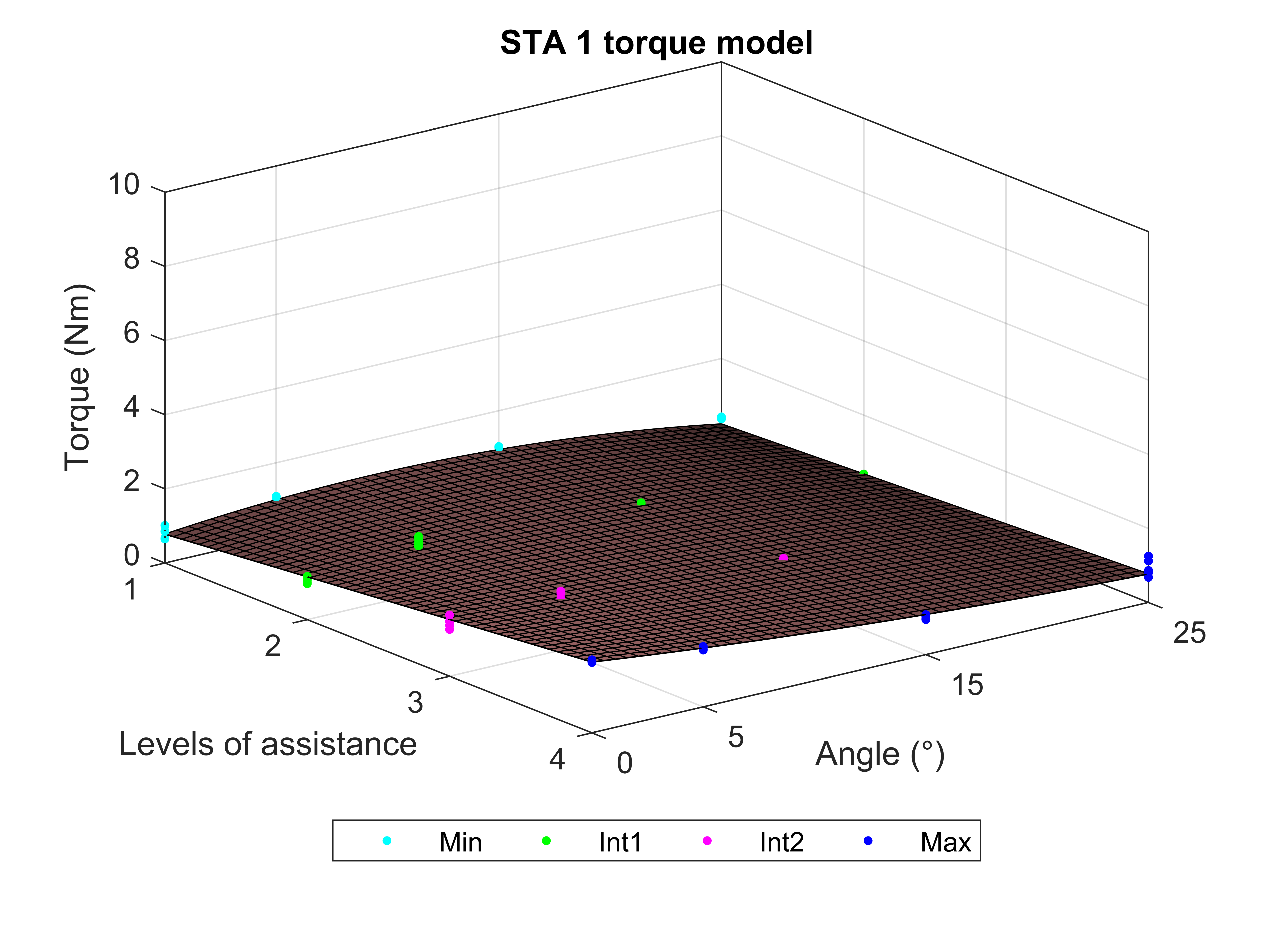

Supplement: Ricard et al. supplementary material 1 — Ricard et al. supplementary material [file S2631717625000088sup001.zip › STA 1.png]

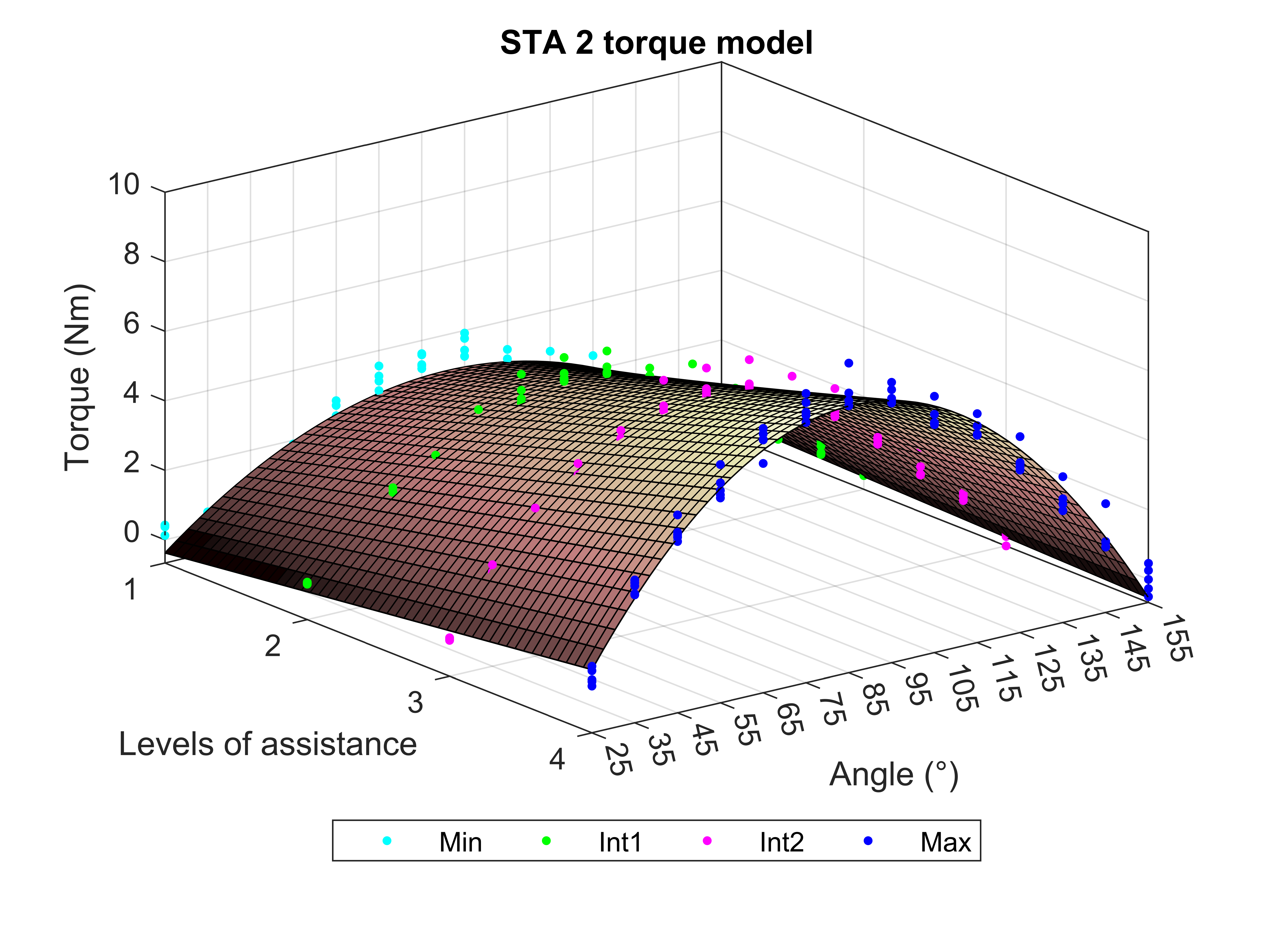

Supplement: Ricard et al. supplementary material 1 — Ricard et al. supplementary material [file S2631717625000088sup001.zip › STA 2.png]

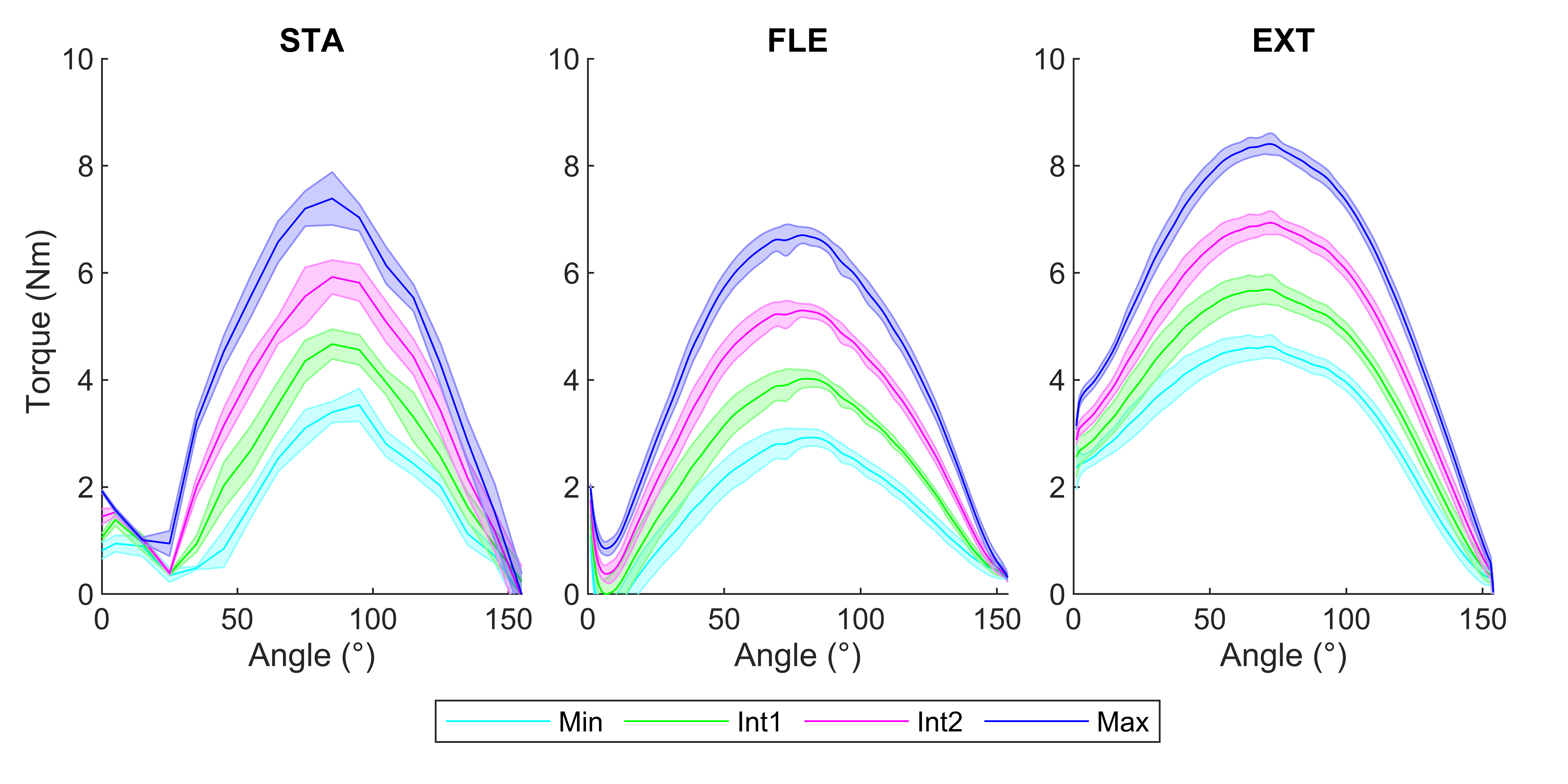

Supplement: Ricard et al. supplementary material 1 — Ricard et al. supplementary material [file S2631717625000088sup001.zip › Torque_angle_Figure 3.png]

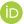

Supplement: Ricard et al. supplementary material 1 — Ricard et al. supplementary material [file S2631717625000088sup001.zip › orcid_logo.pdf]
