# Supplementary material for: Evaluation of the static and dynamic assistive torque of a passive upper limb occupational exoskeleton
Source: Wearable Technol. 2025 Apr 15;6:e19. doi: 10.1017/wtc.2025.8 (PMC12034577; doi:10.1017/wtc.2025.8)
Supplement: Ricard et al. supplementary material 2 — Ricard et al. supplementary material [file S2631717625000088sup002.pdf]

Supplementary material A: correspondence between the opening angle of the exoskeleton and the elevation angle of the operator arm.

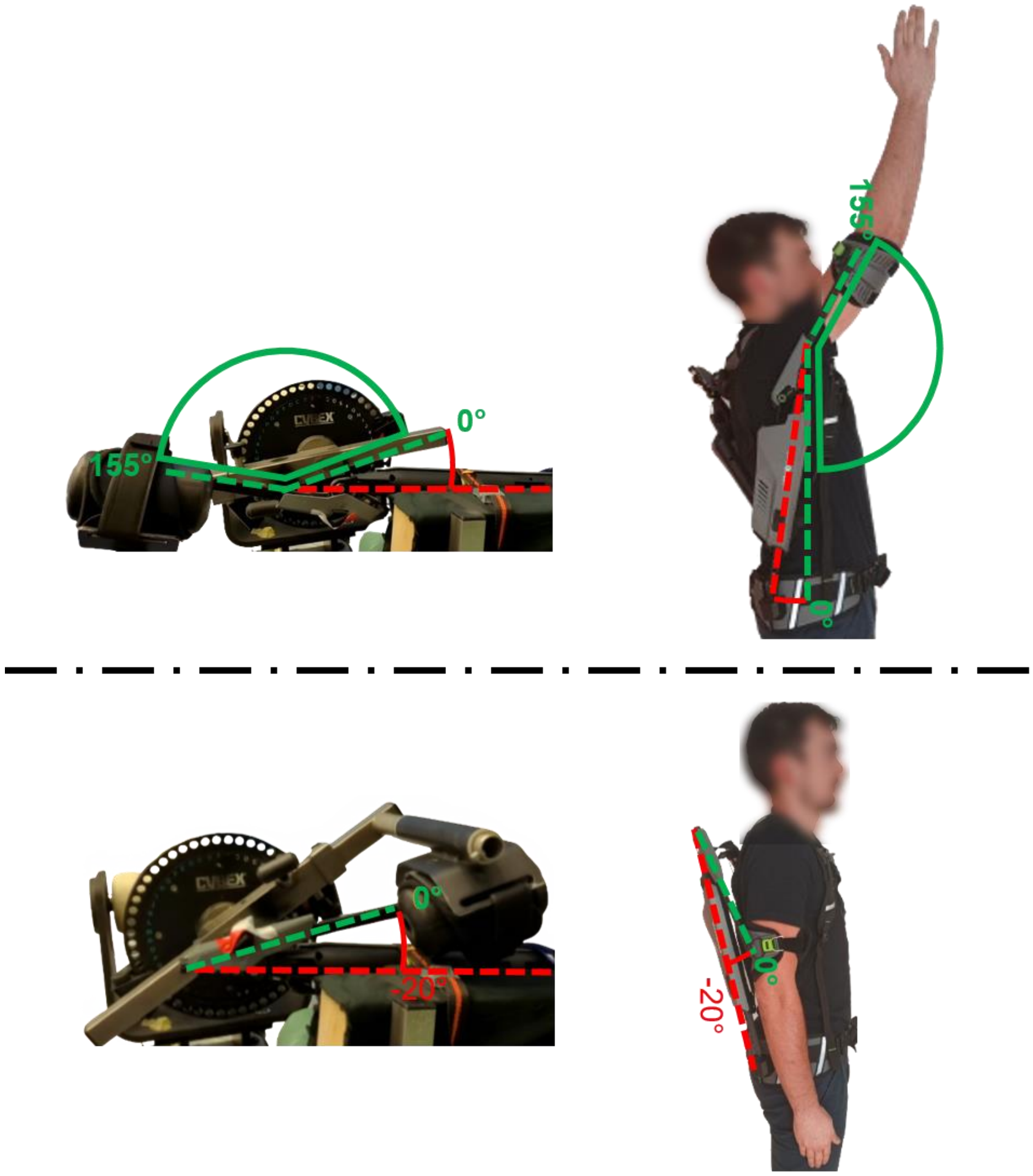

Figure A. Range of motion of the exoskeleton arm hinge joint was set from 0° corresponding to a minimum opening exoskeleton angle (bottom of figure) to 155° corresponding to a maximum opening exoskeleton angle (at the top of the figure). The exoskeleton's opening angle of 0° corresponds to an arm alongside the body for an operator and 155° corresponds to an elevation for the operator's arm.

Supplementary material B: 3D representation of polynomial models of ULE assistive torques.

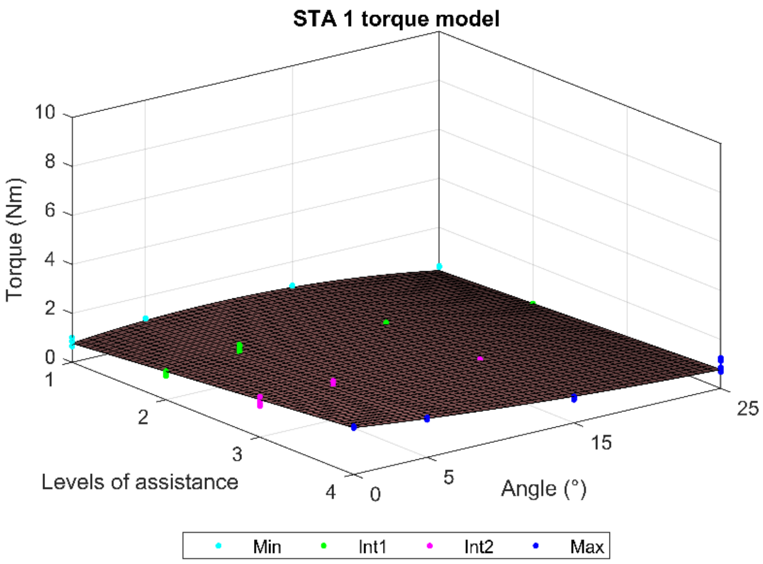

Figure B1. 3D representation of the polynomial modelling of STA 1 corresponds to static tasks on an angular range]0°; 25°]. The equations were f(x,y), where x corresponds to the angular range from 0 to 25° and y the assistance levels between 1 and 4, where 1 is the Min level and 4 the Max level.

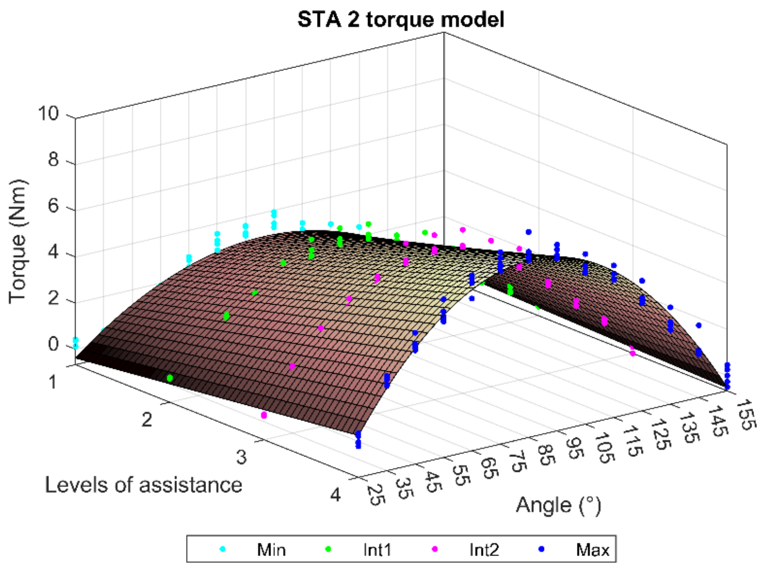

Figure B2. 3D representation of the polynomial modelling of STA 2 corresponds to static tasks on an angular range]25°; 154°]. The equations were f(x,y), where x corresponds to the angular range from 25° to 154° and y the assistance levels between 1 and 4, where 1 is the Min level and 4 the Max level.

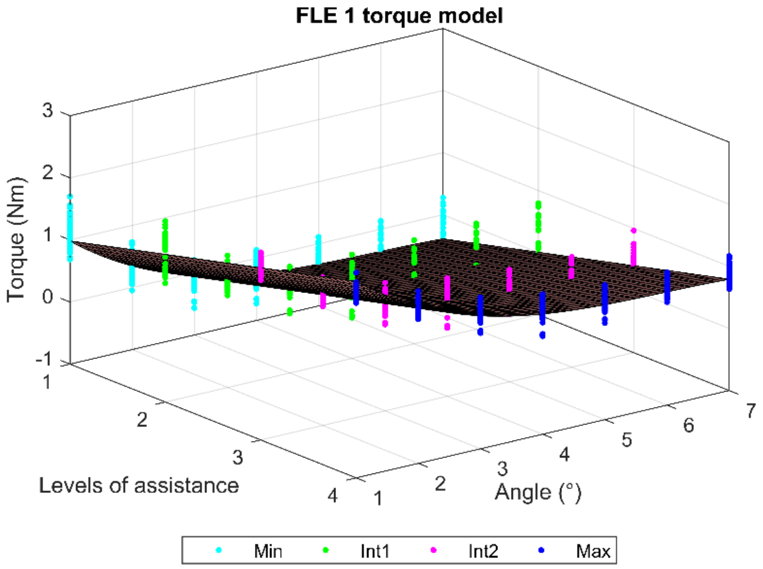

Figure B3. 3D representation of the polynomial modelling of FLE 1 corresponds to dynamic tasks ranging from 20°/s to 240°/s during the exoskeleton arms flexion. The equations were f(x,y), where x corresponds to the angular range from 0° to 7° and y the assistance levels between 1 and 4, where 1 is the Min level and 4 the Max level.

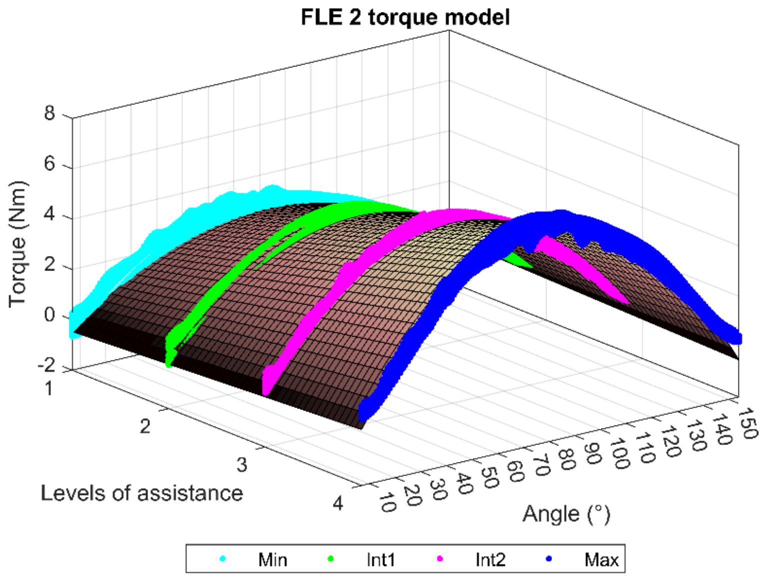

Figure B4. 3D representation of the polynomial modelling of FLE 2 corresponds to dynamic tasks ranging from 20°/s to 240°/s during the exoskeleton arms flexion. The equations were f(x,y), where x corresponds to the angular range from 7° to 154° and y the assistance levels between 1 and 4, where 1 is the Min level and 4 the Max level.

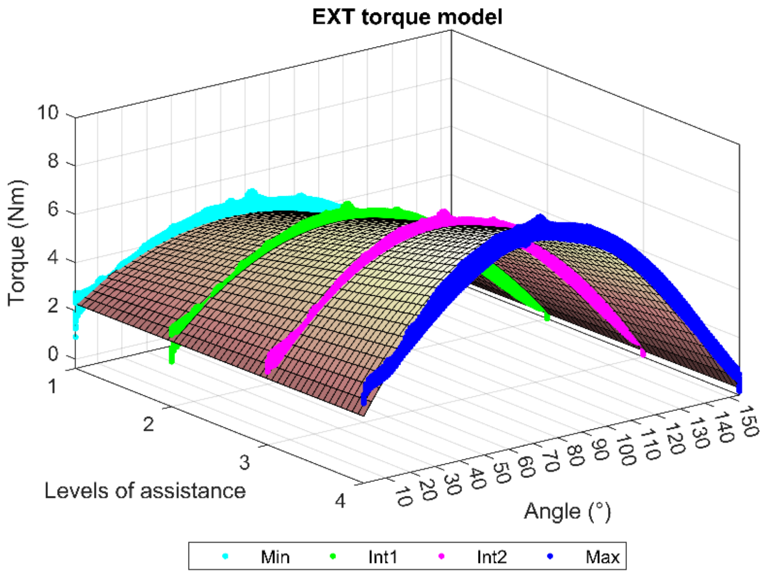

Figure B5. 3D representation of the polynomial modelling of EXT corresponds to dynamic tasks ranging from 20°/s to 240°/s during the exoskeleton arms extension. The equations were f(x,y), where x corresponds to the angular range from 0° to 154° and y the assistance levels between 1 and 4, where 1 is the Min level and 4 the Max level.
